# Breastfeeding satisfaction post hospital discharge and associated factors – a longitudinal cohort study of mothers of preterm infants

**DOI:** 10.1186/s13006-021-00374-4

**Published:** 2021-03-25

**Authors:** Jenny Ericson, Erik Lampa, Renée Flacking

**Affiliations:** 1 Department of Pediatrics, Falu Hospital, Falun, Sweden; 2 Center for Clinical Research Dalarna, Uppsala University, Falun, Sweden; 3 Uppsala Clinical Research Center, Uppsala University, Uppsala, Sweden; 4 School of Education, Health and Social Studies, Dalarna University, Falun, Sweden

**Keywords:** Breastfeeding, Breastfeeding evaluation, Breastfeeding satisfaction, Mothers, Neonatal care, Predictors, Preterm infants

## Abstract

**Background:**

Mothers’ satisfaction with breastfeeding is important for breastfeeding duration but rarely investigated in mothers of preterm infants. The aim of this study was to describe breastfeeding satisfaction and associated factors during the first year in mothers of preterm infants (gestational age < 37 weeks).

**Methods:**

This longitudinal cohort study, based on secondary analysis data from a randomized controlled trial, included 493 mothers of 547 preterm infants. Data on breastfeeding duration and satisfaction, parental stress and attachment were collected at 8 weeks post discharge, and at 6 and 12 months after birth. Breastfeeding satisfaction was measured by the Maternal Breastfeeding Evaluation Scale. Descriptive statistics and linear mixed effect models were used when analyzing the data.

**Results:**

During the first 12 months breastfeeding satisfaction increased in the mean summary scores and points in the dimensions “role attainment” and “lifestyle and maternal body image”. In the dimension “infant growth and satisfaction”, there was an increase in mean points from 6 to 12 months after birth, but not between 8 weeks after discharge and 12 months after birth. The findings also showed that partial and no breastfeeding, higher parental stress, and infant gestational age < 32 weeks were associated with decreased breastfeeding satisfaction. Older maternal age and greater maternal attachment were associated with increased maternal breastfeeding satisfaction. There were no associations between maternal breastfeeding satisfaction and maternal educational level, parity, multiple birth, or maternal birth country other than Sweden, during the first 12 months after birth.

**Conclusions:**

Breastfeeding satisfaction was clearly associated with breastfeeding duration during the first year after birth. Breastfeeding satisfaction may be important to take into account when supporting breastfeeding and when designing interventions to support breastfeeding. Furthermore, these findings highlight the complexity of breastfeeding and emphasize the need for early and good support during neonatal care, so that mothers feel trust in themselves and their infant and in exclusive breastfeeding at discharge and in the first months thereafter.

**Trial registration:**

The randomized controlled trial was registered NCT01806480 with www.clinicaltrials.gov on 2013-03-07.

## Background

Preterm infants (i.e., infants with gestational age, GA < 37 weeks) and their mothers constitute a very vulnerable population in terms of breastfeeding. The rates of breastfeeding are much lower than those in term infants [1, 2], despite the well-known benefits of mother’s milk, especially for preterm infants [3]. Preterm infants are immature in their development, and unlike term infants, the more immature infants cannot be exclusively breastfed at the breast from birth. Subsequently, mothers and infants experience a varying and sometimes long transition period from tube feeding to breastfeeding. How mothers of preterm infants experience the breastfeeding journey varies; for some mothers, breastfeeding is experienced as a positive and smooth journey, while for others, breastfeeding may be burdensome and less positive [4, 5]. The latter kind of experience may be due to a range of factors, e.g., insufficient milk supply, few emotional or practical resources and support, limited person-based support from staff or non-facilitative care routines, and thus result in earlier weaning from breastfeeding [5, 6]. During hospitalization and after discharge, mothers may sustain breastfeeding because they enjoy it and find it pleasurable or because of a cultural norm of breastfeeding and the idea that a ‘good mum breastfeeds’ [7–9].

Across Europe, there are large variations over time, within countries and between countries in the proportion of preterm infants being breastfed. Ericson et al. [1] showed a significant decline in mothers of preterm infants (GA < 37 weeks) breastfeeding exclusively at discharge from Swedish neonatal units within a 10-year period. Bonnet et al. [10] showed, in a large European study in 11 countries, that the average proportion of very preterm (GA < 32 weeks) infants being breastfed at 6 months was 34%, with a range of 25 to 56%. Studies of incidence, duration and trends in breastfeeding are important for quality improvement. High rates of breastfeeding might be seen as synonymous with ‘successful breastfeeding’ and assumed to be mediated by the provision of good care. However, breastfeeding rates only measure one dimension of breastfeeding and may not reflect the mother’s satisfaction with breastfeeding. Breastfeeding satisfaction may be important to assess for the breastfeeding women, health professionals and researchers when evaluating and supporting breastfeeding. Breastfeeding satisfaction might be especially important to measure in the preterm population where the benefits of breastfeeding are more prominent and the mother-infant dyads constitute a vulnerable population in terms of breastfeeding.

Few studies have quantitatively investigated breastfeeding satisfaction or factors associated with breastfeeding satisfaction in mothers of preterm infants. Hence, the aim of this study was to describe breastfeeding satisfaction and associated factors during the first year after birth in mothers of preterm infants (gestational age < 37 weeks).

## Methods

### Design

This study was originally conducted as a randomized controlled trial (RCT) with longitudinal data collection that evaluated a proactive breastfeeding telephone support programme [11–13]. In this paper we have conducted secondary analyses of the longitudinal data.

### Setting

The data in this study were collected between March 2013 and December 2016 as part of the original RCT study. Six neonatal units across Sweden participated in the study.

In Sweden, breastfeeding rates have decreased over the past 15 years. Nevertheless, the initiation rates for breastfeeding are relatively high from an international perspective. However, breastfeeding has been shown to decline rapidly over the first months after birth, especially exclusive breastfeeding [14]. In Sweden, all mothers receive parental benefits, which consist of 480 days per child, 90 of which are reserved for each parent and cannot be transferred. The parental benefit for 390 days is income-based to 80% of the parent’s wages, and for 90 days, the compensation is based on a minimal level [15].

In this study, exclusive breastfeeding was defined as feeding with mother’s milk only but could include medications, fortification and vitamins. Partial breastfeeding was defined as feeding with mother’s milk in combination with infant formula and/or solid food. No breastfeeding was defined as full formula feeding and/or solid food with no mother’s milk intake. The previous 24 h were used as the recall period. The term breastfeeding was used both for breastfeeding at the breast and for mother’s milk feeding regardless of feeding method [16].

### Sample

**Fig. 1:**
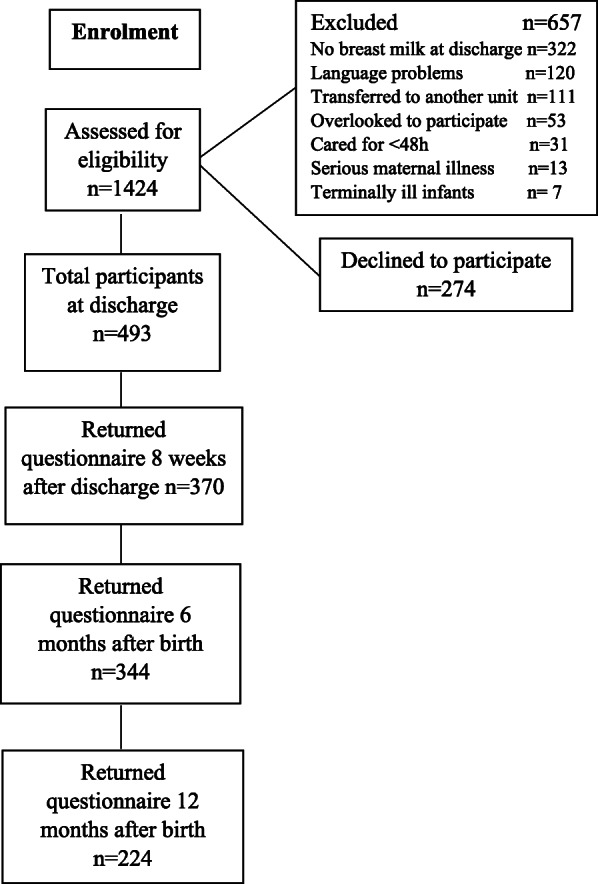
Flowchart of participating mothers

Mothers of preterm infants (gestational age < 37 weeks) who were providing mother’s milk to any extent at discharge were invited to participate in the study. The exclusion criteria included serious maternal medical or psychiatric problems at discharge, language problems that could not be resolved, transfer of the infant to another hospital/unit after discharge, and infants that were terminally ill. In total, 493 mothers of 547 infants contributed data to this study, and a flowchart of eligible, included, excluded and lost to follow-up mothers is shown in Fig. 1. In the study population few mothers fed their infants expressed mother’s milk by bottle. Two mothers exclusively pumped at discharge and had ceased breastfeeding at 8 weeks after discharge. Some mothers stated that they gave their infants expressed mother’s milk by bottle occasionally, but otherwise breastfed direct at the breast. Hence, this study measured breastfeeding satisfaction in mothers who predominantly breastfed their preterm infants directly at breast.

### Data collection and measurements

At each neonatal unit there was a breastfeeding support team consisting of staff from the neonatal unit who informed mothers about the study, asked the mothers for consent, and collected the demographic data close to the discharge. For detailed information on the original RCT study and recruitment see Ericson et al. [17]. All participating mothers signed a written informed consent form after receiving oral and written information and having had the opportunity to ask questions.

Demographic data included the following data for the mothers: age, educational level, parity, country of birth other than Sweden, multiple births and mode of delivery. The following demographic data were collected for the infants: gestational age at birth, birth and discharge weights, small for gestational age status, gender, neonatal illnesses and breathing support during hospitalization, length of stay and breastfeeding at discharge, i.e., exclusive or partial. Follow-up questionnaires were sent by post to the mothers at 8 weeks after discharge, and at 6 and 12 months after birth. The mothers were asked to fill out the questionnaires in relation to how they were feeling at the time regardless of breastfeeding status. The questionnaires included a question on breastfeeding status i.e., exclusive, partial or no breastfeeding and the following validated scales: the Maternal Breastfeeding Evaluation Scale (MBFES) [18], the Maternal Postnatal Attachment Scale (MPAS) [19] and the Swedish Parental Stress Questionnaire (SPSQ) [20]. It took about 20–30 min for the mothers to complete the entire survey.

The MBFES consists of 30 items in the following three dimensions: maternal enjoyment and role attainment, infant satisfaction and growth, lifestyle and maternal body image. Each item is scored on a five-point scale from strongly disagree to strongly agree. A summary score is calculated with a minimum of 30 and a maximum of 150, with higher scores indicating a more positive breastfeeding experience. In our study, the Cronbach’s alpha for the MBFES was 0.93 at all follow-ups. The MBFES is a valid and reliable instrument and was developed to measure aspects of breastfeeding that mothers identify as important in defining successful breastfeeding. The MBFES evaluates the breastfeeding experience, i.e., the mother’s satisfaction with breastfeeding. The three dimensions in MBFES could be used to assess important aspects of breastfeeding satisfaction compared to a single item [18, 21].

The MPAS consists of 19 items, which are scored on a two-, four- or five-point scale and have a total global attachment score with a minimum of 19 and a maximum of 95; higher scores indicate greater maternal-to-infant attachment. In our study, the Cronbach’s alpha was 0.78, 0.76 and 0.74 at 8 weeks after discharge, and at 6 and 12 months after birth for the MPAS, respectively.

Because there is no Swedish version of these two scales, translations were made according to the study by Wild et al. [22], which describes guidelines and standards for the translation and cultural adaptation of patient-reported outcome measures.

The SPSQ, which is a modified Swedish version of the Parenting Stress Index (PSI) [23], consists of 34 items. The items are scored on a five-point scale from strongly disagree to strongly agree. The total score is calculated as the mean of all responses. Higher scores indicate greater perceived parental stress. In our study, the Cronbach’s alpha for the SPSQ was 0.90, 0.89 and 0.90 at 8 weeks after discharge, and at 6 and 12 months after birth, respectively.

The participants’ personal data were obtained and stored in accordance with the European General Data Protection Regulation and the Swedish Ethical Review Act. All collected data were protected against unauthorized access in a secure database with daily back up. The results are presented at the group level, and no individual can be identified.

### Data analysis

As the intervention in the original RCT study had no effect on breastfeeding satisfaction and exclusive breastfeeding [13], all mothers were included in the analyses in the present study, regardless of randomization group. Descriptive statistics of maternal and infant characteristics and the MBFES were calculated and are presented as numbers, percentages, means and standard deviations (*SD*) for normally distributed variables and as medians and interquartile ranges (*IQRs*) for non-normally distributed variables.

To assess associated factors and changes in breastfeeding satisfaction, linear mixed-effects modelling was applied to describe the association between each of the factors and the MBFES summary scores and the scores on each dimension separately over time. The linear mixed-effects model is a useful statistical analysis for longitudinal data as it accounts for the dependence among the repeated measurements on the same individual. It also has an advantage in handling missing outcome values in that an individual can be included in the analysis even if some outcome values are missing. Factors included in the model were maternal age (years), upper secondary school or less/higher education, mothers born in Sweden/other birth country, primipara/multipara, singletons/twins, infant gestational age < 32 weeks/32–36 weeks, breastfeeding at discharge (partial/exclusive) and breastfeeding 8 weeks after discharge and 6 and 12 months after birth (no/partial/exclusive). In the linear mixed-effects models, we used the AR (1) covariance structure with a fixed effect of time. The linear mixed-effects models included repeated measurements 8 weeks after discharge and 6 and 12 months after birth. The results from the linear mixed-effects model analyses are presented with estimates, which should be interpreted as the mean difference in MBFES scores between groups, and 95% confidence intervals (*CIs*) between the compared groups after adjusting for confounders. If the estimated value was positive, the breastfeeding satisfaction level was higher in the reference group; if the value was negative, the breastfeeding satisfaction level was lower in the reference group. The baseline confounders included in the regression models were based on previous research and clinical experience. To test the model, a likelihood ratio test was performed comparing the full model to a null model with a time variable as the only variable. The model fit was assessed visually by comparing the residual distribution to a normal distribution. Calculations were performed with IBM SPSS Statistics for Windows (version 26.0, IBM Corp., Armonk, NY).

## Results

**Fig. 2:**
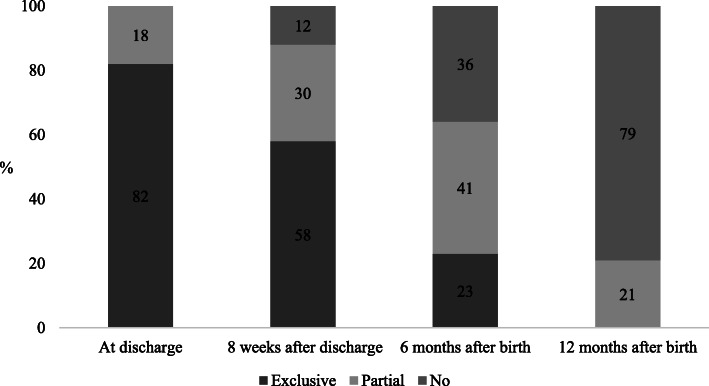
Proportion of mothers breastfeeding (exclusive, partial and no) at each follow-up (discharge, 8 weeks after discharge, and 6 and 12 months after birth), *N* = 493

Numbers and percentages of demographic variables for participating mothers and infants are presented in Table 1. The breastfeeding rates for the sample are presented in Fig. 2. The mean maternal age was 30 years (*SD* ± 5.2). The median gestational age at birth was 34 weeks (*IQR* 2), and the median length of stay was 23 days (*IQR* 21). The mean infant birthweight was 2295 g (*SD* ± 638), and the mean weight at discharge was 2880 g (*SD* ± 473). Three hundred seventy (75%), 344 (70%), and 224 (45%) mothers returned the questionnaire at 8 weeks after discharge and 6 and 12 months after birth, respectively, Fig. 1. Mothers with a lower educational level (*p* < 0.001), mothers not born in Sweden (*p* < 0.001) and mothers who were partially or not breastfeeding 8 weeks after discharge and at a postnatal age of 6 months (*p* = <.001) were less likely to return the questionnaire at all follow-ups.

**Table 1:** Percentages and numbers on demographic variables for participating mothers and their preterm infants

Characteristic	*n* (%)
**Maternal variables**	*N* = 493
Maternal educational level	
Higher education	258 (52)
Upper secondary school or less	235 (48)
Primipara	278 (56)
Mother not born in Sweden	46 (9.3)
Vaginal birth	277 (56)
Multiple birth	52 (11)
**Infant variables**	*N* = 547
Gestational age at birth, median [IQR]	34 [2]
Small for gestational age	43 (8.7)
Male	275 (56)
Neonatal illness during hospital stay^a^	18 (3.7)
Breathing support during hospital stay^b^	233 (47)
Home care	448 (91)

*IQR* Intra quartile range

^a^Bronchopulmonary dysplasia, retinopathy of prematurity, necrotizing enterocolitis, intraventricular heamorrhage, periventricular leucomalacia

^b^Treatment with high flow oxygen nasal therapy, continuous positive airway pressure or a ventilator

### Maternal breastfeeding satisfaction during the first year after birth

The mean and *SD* of the summary scores and each dimension of the MBFES are presented in Table 2. Assessed changes in breastfeeding satisfaction showed increased mean summary scores and points in the dimensions “role attainment” and “lifestyle and maternal body image”. In the dimension “infant growth and satisfaction”, there was an increase in mean points from 6 to 12 months after birth, but not between 8 weeks after discharge and 12 months after birth, Table 3.

**Table 2:** Means and Standard Deviations (*SDs*) of the Maternal Breastfeeding Evaluation Scale (MBFES)

	8 weeks after discharge	6 months after birth	12 months after birth
	Mean ± *SD*	Mean ± *SD*	Mean ± *SD*
MBFES	*n* = 369	*n* = 344	*n* = 224
Summary score	113.7 ± 19.2	111.4 ± 18.7	113.9 ± 18.6
Maternal enjoyment and role attainment	54.9 ± 10.5	54.7 ± 10.9	55.4 ± 10.1
Infant satisfaction and growth	31.4 ± 6.2	28.0 ± 5.7	29.6 ± 6.4
Lifestyle and maternal body image	27.4 ± 6.0	28.6 ± 5.6	28.9 ± 5.6

**Table 3:** Change in breastfeeding satisfaction in mothers (*n* = 387) of preterm infants during the first 12 months postpartum

	Estimate	*95% CI*	*p-value*
Summary score			
8 weeks	− 3.21	−5.29, − 1.13	0.003
6 months	−4.69	−6.21, − 3.18	< 0.001
12 months	ref		
Infant growth and satisfaction			
8 weeks	0.28	−0.57, 1.12	0.52
6 months	−2.38	−3.01, −1.76	< 0.001
12 months	ref		
Maternal enjoyment and role attainment			
8 weeks	−2.09	−3.35, −0.84	0.001
6 months	− 1.89	− 2.80, − 0.98	< 0.001
12 months	ref		
Lifestyle and maternal body image			
8 weeks	−2.62	−3.40, −1.85	< 0.001
6 months	−1.26	− 1.83, −0.68	< 0.001
12 months	ref		

Using linear mixed-effect model analysis presented with estimates, 95% confidence intervals (*95% CIs*) and *p*-*values* for the Maternal Breastfeeding Evaluation Scale summary scores and its three dimensions. The estimate represents the mean difference in points for an individual mother between the follow-ups. Adjusted for the confounder’s education, parity, singleton/twin, gestational age, maternal age, birth country and breastfeeding

### Factors associated with maternal breastfeeding satisfaction

In total, 387 mothers contributed with data in the analysis of the linear mixed effect model of associated factors. Among the variables, there was an association in the model with time only (likelihood ratio test *p* < 0.001). The model fit was acceptable, with the residuals showing a slightly left-skewed distribution. The factors that were associated with a decrease in maternal breastfeeding satisfaction (i.e., summary score) were partial breastfeeding at discharge, partial and no breastfeeding up to 6 months after birth, higher parental stress and infant gestational age < 32 weeks. Factors that were associated with an increase in maternal breastfeeding satisfaction at 8 weeks after discharge, and at 6 and 12 months after birth were higher maternal age and greater maternal attachment. Education, parity, multiple birth and maternal birth country other than Sweden were not associated with maternal breastfeeding satisfaction at 8 weeks after discharge, 6 and 12 months after birth, Table 4. Factors associated with each of the three dimensions are presented in Table 4.

**Table 4:** Factors associated to breastfeeding satisfaction in 387 mothers of preterm infants

	Summary score			Infant growth and satisfaction			Role attainment			Lifestyle and maternal body image		
	Estimate	*95% CI*	*p-value*	Estimate	*95% CI*	*p-value*	Estimate	*95% CI*	*p-value*	Estimate	*95% CI*	*p-value*
Maternal age (per year)	0.64	0.28, 0.99	0.001	0.20	0.09, 0.30	< 0.001	0.21	<−0.01, 0.41	0.05	0.21	0.11, 0.31	< 0.001
Higher education	ref				ref		ref			ref		
Upper secondary school or less	1.98	−1.37, 5.34	0.25	−0.11	−1.11, 0.89	0.83	1.11	−0.86, 3.07	0.27	1.12	0.14, 2.09	0.03
Multipara	ref				ref		ref			ref		
Primipara	0.44	−3.08, 3.95	0.81	−0.27	−1.31, 0.78	0.62	1.20	−0.86, 3.26	0.25	−0.54	−1.56, 0.48	0.30
Other birth country than Sweden	ref				ref		ref			ref		
Born in Sweden	−2.68	−9.26, 3.91	0.43	0.38	−1.59, 2.35	0.71	−2.73	−6.60, 1.13	0.17	−0.40	−2.32, 1.52	0.68
Multiple	ref						ref			ref		
Singleton	2.14	−3.23, 7.50	0.43	1.10	−0.51, 2.71	0.18	0.31	−2.84, 3.46	0.85	0.34	−1.23, 1.91	0.67
Gestational age 32–36 weeks	ref			ref			ref			ref		
Gestational age < 32 weeks	−5.07	−9.60, −0.53	0.03	−2.40	−3.75, −1.05	0.001	−1.54	−4.20, 1.12	0.26	−1.09	−2.41, −0.23	0.10
Breastfeeding at discharge												
Exclusive	ref			ref			ref					
Partial	−10.3	−14.8, −5.79	< 0.001	−4.64	−6.00, −3.28	< 0.001	− 4.75	−7.41, −2.10	< 0.001	−0.44	−1.76, 0.88	0.51
Breastfeeding^a^												
Exclusive	ref			ref			ref			ref		
Partial	−1.93	−3.40, −0.46	0.01	−1.32	− 1.93, − 0.71	< 0.001	−0.69	−1.58, 0.20	0.13	−0.53	−1.09, 0.22	0.06
No	−7.45	−9.60, −5.30	< 0.001	−3.18	−4.05, −2.30	< 0.001	− 3.55	− 4.84, − 2.25	< 0.001	− 2.33	− 3.13, − 1.53	< 0.001
Parental stress, total score (SPSQ)^a^	− 3.29	−5.45, − 1.12	0.003	−0.21	− 1.02, 0.61	0.62	− 014	−1.44, 1.16	0.83	−3.30	− 4.06, − 2.53	< 0.001
Maternal attachment, total score (MPAS)^a^	0.29	0.13, 0.45	< 0.001	0.47	−0.02, 0.11	0.14	0.17	0.07, 0.26	0.01	0.11	0.05, 0.16	< 0.001

Measured at 8 weeks after discharge, 6 and 12 months after birth. Presented with estimates of the difference in mean points, *95%* confidence interval (*CI*) and *p-value* on the Maternal Breastfeeding Evaluation Scale, summary score and the three dimensions

^a^8 weeks after discharge, 6 and 12 months after birth

## Discussion

Our findings showed that mothers who partially breastfed at discharge and at 6 months after birth, had a higher parental stress or a very preterm infant, had a decrease in maternal breastfeeding satisfaction. Further, older mothers and mothers with greater maternal attachment had an increase in maternal breastfeeding. The results also showed that breastfeeding satisfaction increased over time. However, this is probably an effect of the fact that more mothers who were exclusively breastfeeding answered the questionnaires at all follow-ups to a greater extent and thereby increased the mean on the MBFES at the later follow-ups. We speculate that early initiation and supporting breastfeeding may get more mothers to breastfeed for longer and increase breastfeeding satisfaction.

The association between partial breastfeeding and lower satisfaction with breastfeeding is not surprising. It could be assumed that mothers who are partially breastfeeding are less inclined to breastfeed or that their experiences of breastfeeding have been less positive. Early positive experiences and breastfeeding support during neonatal care may facilitate more positive attitudes and higher self-efficacy in breastfeeding, which in turn enables exclusive breastfeeding. Early initiation of breast milk expression and breastfeeding, early and continued skin-to-skin contact, having the means to stay physically close and have privacy to feed in the neonatal unit, and having peers that can guide the mothers, are factors shown to be favourable for breastfeeding [24–27].

Our findings also showed that higher maternal age was positively associated with higher breastfeeding satisfaction. The association is interesting, especially as educational level was not statistically significant; these two factors are usually correlated. Some studies have shown that older mothers are more likely to breastfeed at discharge [28, 29], but the reason why is rarely discussed. Hypothetically, happiness about becoming a mother might be one of the explanations. In a large study conducted by Myrskylä and Margolis [30], young parents (18–23) had a decreasing trend in happiness during the year of birth. Parents aged 23–34 had increased happiness over the year after birth, and mature parents (ages 35–49) were significantly happier than those aged 23–34 during the first year. Furthermore, our findings could potentially be an effect of a lower incidence of mental illness in older women than in younger women. Over the last decade, the proportion of young women with nervousness, apprehension or anxiety has increased in Sweden, and in 2018, 25% of women aged 16–29 had these conditions, while 20% had them in the 30–44 year age group [31]. Ericson et al. [1] have also shown that the prevalence of diagnosed mental illness has increased during the last decade and is significantly associated with breastfeeding at discharge.

Several other factors were associated with breastfeeding satisfaction for example, that greater maternal attachment had a positive association with breastfeeding satisfaction in the summary score and in the dimensions “role attainment” and “lifestyle and maternal body image”. Higher parental stress had a negative association with breastfeeding satisfaction, summary score and the dimension “lifestyle and maternal body image”. Infant gestational age < 32 weeks had a negative association with breastfeeding satisfaction, which may reflect that the immaturity persists even after discharge and influences the maternal breastfeeding experience. These results about attachment, parental stress and infant gestational age show that maternal features were associated with maternal dimensions in the MBFES and with infant features in the infant dimension of the MBFES. This seems logical but also interesting, and it shows the complexity of breastfeeding. Taken together, the results indicate that breastfeeding satisfaction is associated with continued breastfeeding and represents mother’s overall experience of breastfeeding by several dimensions. The mothers’ experience of breastfeeding a preterm infant has previously shown to be complex and an important part of being a mother [5, 9]. Our findings show the importance of considering the emotional experiences, when investigating breastfeeding. A more holistic view on breastfeeding where mothers’ satisfaction is addressed and acknowledged may contribute to new breastfeeding support interventions with improved outcomes on both breastfeeding rates and maternal well-being.

Interestingly, we did not find any associations between the summary score for breastfeeding satisfaction and parity or mothers’ educational level at birth, factors that are often shown to at least influence breastfeeding incidence and duration. It could be that the actual breastfeeding experience does not correlate with these factors, as we have shown. On the other hand, as no previous studies have explored these associations in the preterm population, we need additional studies to substantiate and validate these findings.

The scale we used in this study was the MBFES. This scale was developed by Leff et al. [18] in the early 1990s in response to the need to widen the definition of ‘successful breastfeeding’ to include not only breastfeeding duration but also satisfaction with breastfeeding. In their first qualitative study [32], which formed the basis for the scale, mothers described ‘successfulness’ as “working in harmony”; breastfeeding is an interactive process that results in mutual satisfaction of maternal and infant needs. One could argue that our findings about the evident association between exclusive breastfeeding and breastfeeding satisfaction is a matter of “of course,” i.e., mothers who exclusively breastfeed fulfil the expectations of a norm and subsequently become ‘proud’ or satisfied with the accomplishment. However, with the dimensions included in the MBFES, we believe that the scale captures the holistic experience of breastfeeding and measures harmonious, attuned, and pleasurable breastfeeding in mothers who predominantly breastfeeds their preterm infants directly at breast.

### Limitations

The MBFES has not been validated and adapted for mothers of preterm infants. For example, one question states, “The baby had trouble with breastfeeding at first”, which includes almost all preterm infants during the first breastfeeding period. There is a need for additional research to re-assess the scale according to new trends and care, in which different groups of mothers and their infants can participate in different contexts and with different breastfeeding progressions. Swedish neonatal units are favorable for breastfeeding because of parental leave benefits, parental presence neonatal units, high frequency of skin-to-skin contact, and free of charge access to breast pumps, consequently the generalizability of the present findings could be limited [33]. Another limitation of this study is that it is an observational study in which causality generally cannot be established. The baseline confounders included in the mixed models were based on previous research and clinical experience, but we cannot rule out that one or more important confounders was not included. Many mothers were not eligible for participation in the study as they did not breastfeed at discharge (*n* = 322, 23%). Mothers with higher education, born in Sweden and who breastfed exclusively responded to a greater extent to the questionnaires during the later follow-ups. Altogether, the criteria for exclusion and the response rates may affect the results. The results should be interpreted with these limitations in mind. Strengths of this study are the longitudinal design, the assessment of several potential predictors of breastfeeding satisfaction and the large sample size. Furthermore, the use of the linear mixed effect models that handle missing data i.e., the mothers did not need complete data at all follow-ups to be included in the model.

## Conclusions

Lower breastfeeding satisfaction was associated with partial breastfeeding, parental stress and lower gestational age, and higher breastfeeding satisfaction was associated with older maternal age and greater attachment through the first year after birth in mothers of preterm infants. These findings highlight the complexity of breastfeeding. Early and good support during neonatal care and continued support thereafter may enhance mothers trust in themselves, in their infant, and in their breastfeeding. Additionally, breastfeeding satisfaction may be an important measure to take into account when supporting breastfeeding and when designing interventions to support breastfeeding.

## Data Availability

The datasets generated and analyzed during the current study are not publicly available for ethical and legal reasons.

## Abbreviations

MBFES: Maternal Breastfeeding Evaluation Scale
MPAS: Maternal Postnatal Attachment Scale
RCT: Randomized Controlled Trial
SPSQ: Swedish Parental Stress Questionnaire
